# Explicit (Not Implicit) Attitudes Mediate the Focus of Attention During Sentence Processing

**DOI:** 10.3389/fpsyg.2020.583814

**Published:** 2020-12-23

**Authors:** Oleksandr V. Horchak, Margarida Vaz Garrido

**Affiliations:** Centro de Investigação e Intervenção Social, Iscte-Instituto Universitário de Lisboa, Lisbon, Portugal

**Keywords:** situation model, protagonist dimension, comprehender characteristics, language comprehension, environmental attitude

## Abstract

Many studies showed that comprehenders monitor changes in protagonists’ emotions and actions. This article reports two experiments that explored how focusing comprehenders’ attention on a particular property of the protagonist dimension (e.g., emotional or action state) affects the accessibility of information about target objects mentioned in the sentence. Furthermore, the present research examined whether participants’ attitudes toward the issues described in the sentence can modulate comprehension processes. To this end, we asked participants to read sentences about environmental issues that focused comprehenders’ attention on different mental and physical attributes of the same entities (protagonists and objects) and then self-report their own thoughts on the topic of environment by responding to the items assessing their environmental awareness. Importantly, we manipulated the task requirements across two experiments by administering a self-report task (Experiment 1), which required the participants to rate the seriousness and the frequency of the problem mentioned in a sentence; and administering a sentence-picture verification paradigm (Experiment 2), which required the participants to merely indicate if the object depicted in the picture (related to a certain environmental problem) was mentioned in the preceding sentence. The results of these experiments suggest that the focus of a sentence on the environmental problem (rather than the protagonist’s emotion and action) enhances the accessibility of information about environmental issues (e.g., plastic garbage); that the comprehender’s level of environmental awareness influences one’s attention during sentence processing; and that comprehender characteristics significantly modulate comprehension processes only when the measures tap into explicit (and not implicit) processes.

## Introduction

Since the introduction of the construct of mental or situation model (Kintsch and van Dijk, 1978; Johnson-Laird, 1983), the notion that language comprehension requires the construction of mental representations of the agents, objects, locations, events, and actions described in a text has become a mainstream position adopted by many researchers in the areas of linguistics, psychology, and more generally, cognitive science. This perspective has generated many interesting lines of research that allowed us to come closer to answering the question how comprehenders understand the meaning of language. According to Zwaan et al.’s (1995a) event-indexing model, comprehenders monitor information described in a story at the event level, whereby each event can be indexed on the following five dimensions: time (Magliano and Schleich, 2000; Rinck et al., 2003), space (Glenberg et al., 1987; Morrow et al., 1989; Rinck and Bower, 2000), causation (Trabasso and Sperry, 1985; Trabasso and Suh, 1993), motivation (Lutz and Radvansky, 1997; Rinck and Bower, 2004), and protagonists and objects (Myers et al., 1994; Cook et al., 1998). Thus, from a situation model perspective comprehenders create a coherent representation of story events by means of the immersive experience of the story world, which is very similar to how people track and process events in real-life (Zwaan, 2004; Mulcahy and Gouldthorp, 2016).

In their review of situation model research, Zwaan and Radvansky (1998) argued that such entities as protagonists and objects form the “meat” of situation model construction. These entities, in turn, are defined by their corresponding properties (e.g., physical and mental attributes) that are the most relevant for meaning-making processes during language comprehension. Many experiments confirmed compellingly this claim. With regard to such properties as character traits, Albrecht and O’Brien (1993) showed that participants’ reading times were slower when they read about a vegetarian ordering a hamburger, thus suggesting that comprehenders store the protagonists’ traits in memory during reading. With regards to emotional states, Gernsbacher et al. (1992) demonstrated that critical sentences were read more slowly when they contained an emotion word that is inconsistent with the emotional state implied by the story (e.g., processing the word “pride” after reading how someone was fired). Similarly, Komeda and Kusumi (2006) discovered that discontinuities in the emotional dimension (e.g., *worry–relief* vs. *relief–relief*) lead to significant increases in reading times, further implicating a constant situation model updating during reading. With regards to goals, Long et al. (1992) tested and confirmed a hypothesis that understanding a sentence like “The dragon kidnapped the daughters” leads to the generation of superordinate goal inferences (e.g., the dragon will eat the daughters). Rinck and Bower (2004) found that objects relevant to the current goal (e.g., a xerox machine when reading about the action of printing) were more accessible for readers than those that are irrelevant. Thus, altogether these results support the claim that the protagonists, objects, and their properties are the core around which situation models are created.

The above studies on the importance of entities and their properties (e.g., emotional states, action) made significant headway in assessing the influence of dimensional focus during situation model construction. As reviewed above, one common finding in this literature is that reading times increase when there are some discontinuities along a particular single property, such as, for instance, mismatching emotion or goal-irrelevant action (see Therriault and Rinck, 2007, for discussion). This finding is consistent with a processing load hypothesis of the Event-indexing model (Zwaan et al., 1995a) and an Event Segmentation theory (Zacks et al., 2007), which suggest that comprehenders should find it more difficult to integrate the current event into their situation model when there are few indices that are shared between the past and present events. Therefore, increased reading times reflect the fact that readers need to update a situation model to be able to incorporate new information that mismatches, even if partially, the situation described by the previous information.

Related to the idea of how readers guide their focus of attention during language comprehension is the research on the impact of situation models on memory retrieval. A series of experiments (Radvansky and Zacks, 1991; Radvansky et al., 1993; Sohn et al., 2004) used a so-called *fan effect* paradigm (Anderson, 1974) to demonstrate that response times are increased as a function of the number of associations with a concept stored in memory. Among the most popular of such experiments are those in which participants have to memorize sentences that describe objects in either a single-location condition or a multiple-location condition. A fan effect (i.e., an increase in retrieval time) is usually observed in a speeded-recognition test when a single object is described as being in several locations (e.g., “The painting is in the hotel,” “The painting is in the store,” “The painting is in the store”) than when different objects are described as being in the same location (e.g., “The painting is in the hotel,” “The wardrobe is in the hotel,” “The bed is in the hotel”). Such results line up with arguments that in a multiple-location condition different situation models are activated that interfere with a comprehender’s ability to retrieve the desired mental representation (Radvansky et al., 1998).

Although the role of the aforementioned inhibitory processes has been at the focus of research in cognitive psychology for quite some time (see Radvansky, 1999, for discussion), significant issues remain to be addressed. The first question concerns the extent to which focusing comprehenders’ attention on a particular property of the entity affects the accessibility of objects mentioned in the sentence. As discussed before, previous empirical research has mostly examined how the accessibility of information is affected by the entity’s single property (e.g., emotion or action). While this research has deepened our understanding of the specifics of each individual property, it is somewhat unclear how multiple properties of the same entity determine how facts are integrated into a situation model. For example, if the sentences (1) “John *noticed* the garbage on the beach,” (2) “John *got upset* with the garbage on the beach,” and (3) “John *picked up* the garbage on the beach” are processed, a comprehender is likely to represent these differently. This is the case because all of these sentences share the same concept (i.e., garbage on the beach) with one fundamental difference: the sentence (1) – places emphasis on the environmental issue, the sentence (2) – on the protagonist’s emotional state and the sentence (3) – on the protagonist’s action. Therefore, the critical research question is: will properties of the model that are currently at focus receive high activation, so that information about them will impair accessibility to the objects (i.e., garbage) mentioned in the sentence? This is a fairly straightforward extension of previously discussed research showing that readers infer information related to emotion and action, thus making it highly accessible in memory.

The second way in which we hope to advance our understanding of the relation between situation model construction and language processing is by examining whether the participants’ attitudes and sensitivity toward the issues described in the sentence can modulate comprehension processes. While previous research related to situation model construction specified how events occur across space and time, involve protagonists and objects, and have intentional and causal structures, few empirical attempts have been made to explore how language processing mechanisms interact with the characteristics of the comprehender (see Knoeferle, 2019, for an in-depth discussion). This is unfortunate as without integrating this kind of information into a situation model there is no way to know whether language processing can vary as a function of comprehender characteristics. Consider, for example, a situation in which environmental problems such as air pollution, water pollution, and global warming are discussed. There is a robust and well-established literature showing that people considerably differ in their pro-environmental attitudes. For some people, environmental awareness is important to the extent that they feel a responsibility to act (Liu and Leiserowitz, 2009). For others, in contrast, environmental awareness is less important, so that they do not think there is a need to minimize the negative impact of people’s actions on the surrounding environment (Kennedy et al., 2009). Given this difference in attitudes, it is reasonable to suggest that a comprehender may adopt a somewhat different interpretation of a sentence depending on the level of his/her environmental awareness. The experiments to be reported test this hypothesis, specifically examining the influence of the comprehender’s environmental awareness upon the accessibility of environment-related information in different conditions of attentional focus (i.e., object-focus vs. emotional state vs. action state) during sentence comprehension.

Documenting the contributions of participants’ attitudes in situation model construction could provide a more comprehensive understanding of the language comprehension processes. However, social cognition research suggests that it could be useful to distinguish between tasks that tap into unconscious automatic responses and tasks that tap into conscious intentionally edited responses when assessing the mediating role of attitudes (Fazio and Olson, 2003; Hofmann et al., 2005). Meissner et al. (2019) argued that tests of implicit measures of attitudes (i.e., tests that obscure the content of measurement) like Implicit Association Test (Greenwald et al., 1998) aim to measure evaluation (“liking”) instead of motivation (“wanting”), thus suggesting that the superiority of these tests over self-report measures is questionable. Similarly, Wilson et al. (2000) suggested that explicit and implicit measures oftentimes reflect different evaluations of the same object, given that implicit tests measure highly stable, old representations and explicit tests measure more recently acquired, new representations. This suggests that previously formed representations may still be accessible in memory when new contradicting information about a concept is acquired. As noted by Gawronski and De Houwer (2014), the consequence of this is that people may have two distinct attitudes toward the same concept: the previously acquired “implicit” attitude that gets activated automatically upon encountering a relevant stimulus; and a more recently acquired “explicit” attitude that requires some controlled processes (conscious effort) to be successfully retrieved from memory. The latter point is of particular interest because it is possible that task demands may determine whether the effects of comprehender characteristics and, more generally, of the socially interpreted context are detected: if people consider themselves to be pro-environmentally committed but still find it hard to live up to their ideals, then there remains a possibility that only direct measurement procedures (i.e., self-reports) may capture people’s pro-environmental attitudes during language comprehension. Thus, one of the aims of this research was to test this possibility by using two different tasks: a sentence-picture verification task (akin to “implicit” measure) and a self-report (akin to “explicit” measure).

## The Present Research

On the basis of considerations outlined in the previous section, it seems plausible that comprehender characteristics may be a modulatory factor in language comprehension depending on whether the task taps into “explicit” or “implicit” attitudes. Therefore, in the present research we assessed the impact of attitudes on the accessibility of objects in different conditions of attentional focus while participants were performing two different tasks. In Experiment 1, we used a direct self-report measurement procedure, where participants had to read a sentence describing a certain environmental problem and then rate the seriousness and the frequency of the problem mentioned in the just-read sentence on a 10-point Likert scale. The focus of participants’ attention in the critical sentences was manipulated by the critical verb used to describe an event (e.g., “John *noticed* the garbage on the beach,” “John *got upset* with the garbage on the beach,” “John *picked up* the garbage on the beach”). In Experiment 2, in contrast, we used an indirect measurement procedure, where participants read the same sentences as in Experiment 1, except that their task was to decide as quickly as possibly whether or not the subsequently presented pictured object (e.g., plastic garbage) was mentioned in the sentence. The just-mentioned sentence-picture verification paradigm from Experiment 2 should considerably reduce participants’ ability to control their responses given social desirability, and hence the impact of attitudes on participants’ responses may be considered resource-independent and unconscious (Gawronski and De Houwer, 2014). At the end of both Experiments 1 and 2, we assessed participants pro-environmental attitudes by asking them to respond to a 30-item Environmental Attitudes Inventory (Milfont and Duckitt, 2010), which was validated for Portuguese population (Domingues and Gonçalves, 2018).

## Experiment 1

In Experiment 1, we measured participants’ ratings of the seriousness and frequency of the problem mentioned in the sentence on 10-point Likert scale (1 = *Not serious at all* and 10 = *Very serious*). We predicted that the ratings will be higher in the “object-focus” condition (e.g., “John *noticed* the garbage on the beach”) than in the “emotion-focus” (“John *got upset* with the garbage on the beach”) and “action-focus” (“John *picked up* the garbage on the beach”) conditions. Such a prediction follows from previous studies showing that readers assign high priority to the protagonist’s mental and physical states (see Therriault and Rinck, 2007; Bower, 2008, for a discussion), which, as a consequence, may interfere with the retrieval of relevant information. Rinck and Bower (2004) proposed that attentional focus during reading may be compared to a spotlight shining into a dollhouse, where the situation model is an inner stage that comprehenders construct in working memory. By using this analogy, when a target object is in focus (i.e., environmental problem), it is as if the spotlight is shining on it, thus increasing its availability in memory relative to when a protagonist’s emotion or action are in focus. Consequently, when information is at a higher level of availability (as is the case in the “object-focus” condition), participants’ ratings of the seriousness and the frequency of the problem mentioned in the sentence should increase. Furthermore, if comprehenders’ attitudes mediate language comprehension processes, then we would expect to see an interaction such that the ratings in the “object-focus” condition should be higher only when taking into account the scores of the participants who are more environmentally concerned. Experiment 1 was designed to test these predictions.

### Method

#### Sample Size and Ethical Requirements

We conducted a Power analysis in G^∗^Power to determine the necessary number of observations for both Experiments 1 and 2. A power analysis was done using an effect size (*d* = 0.31, alpha level of 0.05, and a power of 0.80) from the study of Zwaan et al. (2002), which used the same sentence-picture verification paradigm as that used in Experiment 2 of the present research. According to Brysbaert and Stevens (2018), the typical effect size in many psycholinguistic experiments is even smaller (*d* = 0.10 or *d* = 0.20). Therefore, we determined our sample size by running a power analysis on a repeated measures ANOVA, a power of 0.80, an alpha level of 0.05, and a small ($\eta_{\text{p}}^{2}$ = 0.02) effect size. The results of this analysis suggested that we would need 99 participants to find an effect if it existed. Of note, this sample size is comparable to the thematically related studies of Therriault et al. (2006) and Bailey et al. (2017), which studied the influence of dimensional focus during situation model construction using different methods. To account for low accuracy, compliance with the task requirements, or data saving errors, we always attempted to collect data from at least 110 participants.

The study was carried out in accordance with the World Medical Association’s Declaration of Helsinki. In line with the ethical guidelines of the host institution, participants from both Experiments 1 and 2 gave informed consent prior to participation and were fully debriefed about the purpose of the study upon completion.

#### Participants

One hundred and ten native Portuguese-speaking participants (*M*_age_ = 27.90, *SD*_age_ = 10.47; 49 males) were recruited via Prolific Academic (Palan and Schitter, 2018) by using the following prescreening criteria: Country of Birth = Portugal; Country of Residence = Portugal, and First (Native) Language = Portuguese. Participants were compensated at a rate of £5.05 (British pounds) per hour.

#### Materials

Seventy-two sentences were created: 18 experimental sentence triads and 18 filler sentences. The experimental sentence triads “invited” participants to attend their attention to the environmental issue (e.g., “John *noticed* the garbage on the beach”); the protagonist’s emotional state (e.g., “John *got upset* with the garbage on the beach”); and the protagonist’s action (e.g., “John *picked up* the garbage on the beach”). Thus, we varied the proximity of target objects to the focus of attention by using verb information. Filler sentences were of the same format as experimental sentences with a fundamental difference: they described less environmentally serious events (e.g., “John brushed his teeth with the bamboo toothbrush”), positively laden events (e.g., “John liked his new bottle made of recycled glass”), and emotionally neutral (“John examined his bicycle”) events. The purpose of these filler sentences was to discourage participants from selectively paying attention to more serious environmental problems and, consequently, regulate their responses in line with social desirability (Gawronski and De Houwer, 2014). The list of critical sentences used in Experiments 1 and 2 is provided in Supplementary Appendix A.

A short 36-item version of the Environmental Attitudes Inventory was used (Milfont and Duckitt, 2010) to calculate a mean score of participants’ environmental awareness. This set of 7-point scales assesses environmental attitudes that underlie individual’s behavior toward the environment on such dimensions as preservation and utilization. Domingues and Gonçalves (2018) and Domingues et al. (2019) assessed the structure of the Portuguese version of the Environmental Attitudes Inventory of Milfont and Duckitt (2010) and found that scales 5 (confidence in science and technology) and 12 (support for population growth policies) did not reflect participants’ attitudes toward the environment in Portugal. Thus, we removed these scales and calculated the mean score of environmental awareness from the remaining 30 items (see Supplementary Appendix B, for the list of all items used).

#### Design and Procedure

Three lists of stimuli were created to counterbalance items and conditions, so that the same items that appeared in one sentence condition for some participants were in the different sentence conditions for other participants. Each participant was randomly assigned to one of the lists. This produced a 3 (sentence condition: object-focus, emotion-focus, action-focus) × 3 (list) design, with sentences being a within-participants factor and list a between-participants factor. As list was not something that was actually manipulated, we did not include it as a factor in the reporting of statistical analyses. Each participant was exposed to 18 experimental sentences (i.e., six sentences for each sentence condition) and 18 filler sentences, and then provided their responses to the 30 items of the Portuguese version of the Environmental Attitudes Inventory.

Stimulus presentation was controlled in Qualtrics Survey Software. In the first part of the experiment, participants read sentences, which were presented in a random order, about some environmental problem (one sentence at a time) and rated the seriousness and the frequency of the problem mentioned in the just-read sentence on a 10-point scale from 1 (*Not serious/frequent at all*) to 10 (*Very serious/frequent*). In the second part of the experiment, participants indicated their level of agreement with statements from the Portuguese version of the Environmental Attitudes Inventory on a 7-point scale from 1 (*Completely disagree*) to 7 (*Absolutely agree*).

#### Statistical Analysis

All statistical analyses were performed within the R programming environment version 4.0.0 (R Core Team, 2020) and several R packages. R Markdown files were used to generate code and the analyses were “knit” into html files. We fitted an ordinal mixed-effects model (cumulative link mixed model) with random effects of participants and items using an “ordinal” R package (Christensen, 2019). This model is optimal for Likert-type data as it allows to predict an ordinal dependent variable given one or more independent variables. Furthermore, this model permits to simultaneously account for two random variables in our design (participants and items), which is more powerful than separate by-participants (usually denoted as *F*_1_) and by-items (usually denoted as *F*_2_) analyses (Brysbaert and Stevens, 2018). The full or “maximal” (Barr et al., 2013) model contained sentence condition, environmental concern score, as well as the interaction between the two as fixed effects; a by-participants random slope for sentence condition; and an intercept for participants and items. No varying slopes were considered for items or environmental awareness as the test stimuli for these two factors were never repeated (Barr et al., 2013). To make interpretation of parameter estimates easier when testing an interaction between a continuous variable (environmental concern score) and a categorical variable (sentence type), environmental concern scores were standardized by subtracting the mean and dividing by the standard deviation for analysis. We used the default R “treatment” coding scheme, where each level of the categorical variable is contrasted to a specified reference level. In the present research, the “object-focus” condition was set as a reference category. Given that the interpretation of main effects is affected by the presence of an interaction term when fitting models using treatment contrasts (i.e., lower order terms reflect the effect of one variable at the specific level of the other independent variable; see Singmann and Kellen, 2020, for discussion), in both Experiments 1 and 2 we approached the analysis of data in the following way. First, we used a likelihood ratio test that compared the likelihood of a model with the interaction term to the likelihood of a model without it to determine whether the overall interaction between variables was significant. If a likelihood ratio test was significant, then we reported the results of the model involving an interaction term, which contained two fixed effects (i.e., sentence condition, environmental concern) and two interaction terms comparing each of the non-referent levels (“emotion-focus” condition, “action-focus” condition) to the referent level (“object-focus” condition). If a likelihood ratio test was not significant (i.e., the presence of an interaction was not established), then we removed the non-significant interaction term from the model (to facilitate the interpretation of lower-order terms) and reran the analysis with fixed effects only (i.e., sentence condition, environmental concern). Following the guidelines of Meteyard and Davies (2020), detailed results of the final models from Experiments 1 and 2 are provided in Supplementary Appendices C and D, respectively.

## Results and Discussion

**FIGURE 1:**
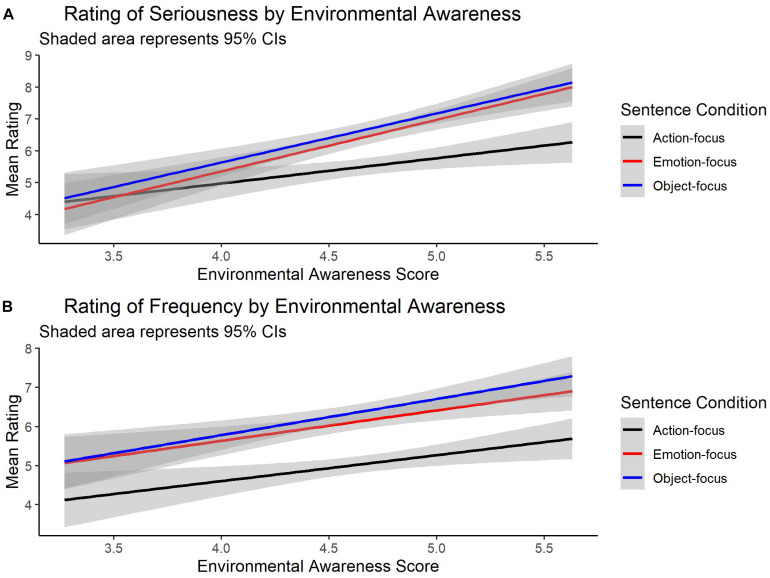
Graphs **(A,B)** present regression lines or “lines of best fit” for rating of seriousness and frequency as a function of environmental awareness.

The data of major interest are provided in Figure 1^1^. With regards to the ratings of seriousness, a likelihood ratio test of the “maximal” model with fixed effects of sentence condition, environmental concern, as well as the interaction between the two against the model with fixed effects of sentence condition and environmental concern revealed a significant difference between the models [χ^2^(2) = 9.10, *p* = 0.01], thus suggesting that the interaction between sentence condition and environmental concern was significant. The results of the “maximal” model showed a significant effect of “action-focus” condition (estimate = −0.79, *SE* = 0.11, *z* = −7.00, *p* < 0.001, odds ratio = 0.45), reflecting the fact that ratings in this condition were significantly lower (*M* = 5.49, *SD* = 3.38) than in the “object-focus” condition (*M* = 6.65, *SD* = 3.15). There was no significant effect of “emotion-focus” condition (estimate = −0.14, *SE* = 0.12, *z* = −1.17, *p* = 0.244, odds ratio = 0.87) despite the fact that ratings in this condition were also lower (*M* = 6.42, *SD* = 3.24) than in the reference (i.e., “object-focus”) condition. Furthermore, as expected, there was a significant effect of participants’ environmental concern (estimate = 0.52, *SE* = 0.15, *z* = 3.56, *p* < 0.001, odds ratio = 1.68), which reflects the fact that participants with high environmental concern rated environmental problems as more serious than those with low environmental concern. Finally, as illustrated in Figure 1A, there was a significant interaction between environmental concern and “action-focus” condition (estimate = −0.24, *SE* = 0.11, *z* = −2.13, *p* = 0.033, odds ratio = 0.79), but no significant interaction between environmental concern and “emotion-focus” condition (estimate = 0.09, *SE* = 0.12, *z* = 0.72, *p* = 0.470, odds ratio = 1.09). This result suggests that action-focused sentences received lower ratings of seriousness than object-focused sentences only from participants with higher concern over environmental issues.

With regards to the rating of frequency, a likelihood ratio test of the “maximal” model with fixed effects of sentence condition, environmental concern, as well as the interaction between the two against the model with fixed effects of sentence condition and environmental concern showed no significant difference between the models [χ^2^(2) = 1.44, *p* = 0.487], thus suggesting that the interaction between sentence condition and environmental concern was not significant. Therefore, the “simplified” model without an interaction term was used in the reporting of results. The results of the “simplified” model revealed a significant effect of “action-focus” (estimate = −1.17, *SE* = 0.11, *z* = −10.39, *p* < 0.001, odds ratio = 0.31) and “emotion-focus” (estimate = −0.23, *SE* = 0.10, *z* = −2.24, *p* = 0.025, odds ratio = 0.80) conditions. That is, ratings in the “action-focus” (*M* = 5.04, *SD* = 2.75) and “emotion-focus” (*M* = 6.15, *SD* = 2.62) conditions were lower than in the referent “object-focus” (*M* = 6.39, *SD* = 2.69) condition. There was also a significant effect of participants’ environmental concern (estimate = 0.27, *SE* = 0.12, *z* = 2.33, *p* = 0.020, odds ratio = 1.31), which suggests that, as expected, more environmentally concerned participants rated the problems described in the sentence as more frequent than less environmentally concerned participants.

To summarize, the results from Experiment 1 suggest that participants’ responses depended on two independent variables: attentional focus and environmental concern. Ratings of the seriousness and frequency of an environment issue (e.g., plastic rubbish, raised water level, mud from rains) were lower when sentences focused on the protagonist’s emotion or action rather than the sentence object (environment issue). Furthermore, participants with higher concern over environmental issues (from a test of attitudes) gave higher ratings. Thus, both protagonist and comprehender characteristics affected the way participants focused their attention during sentence comprehension. Contrary to our prediction, however, ratings for object-focused sentences were not always higher for participants with greater environmental concern. With regards to ratings of seriousness, only half of our prediction was validated: object-focused sentences produced higher ratings for those with greater environmental concern when compared to the action-focused sentences. However, this pattern was not repeated when ratings for object-focused sentences were contrasted with ratings for emotion-focused sentences. That is, the differences in ratings between object-focused and emotion-focused sentences were almost identical when taking into account the ratings of participants with both lower and greater environmental concern (Figure 1A). With regards to ratings of frequency, there was very little evidence of one variable (environmental concern) depending on the other (sentence condition): object-focused sentences always produced the highest ratings, whereas action-focused sentences produced the lowest ratings, with emotion-focused sentences (almost always) falling roughly in between (Figure 1B). Furthermore, in contrast to ratings of seriousness, object-focused sentences did produce significantly higher ratings when compared to emotion-focused sentences for the ratings of frequency. When put alongside evidence for the causal role of comprehenders’ characteristics for the rating of seriousness (as evidenced by a significant interaction between action-focused sentences as compared to the object-focused sentences), it appears that unique comprehenders’ characteristics, such as environmental attitudes, affect attentional focus to the extent to which a self-report measure asks participants to explicitly evaluate their attitudes to the problem described in the sentence. Presumably the effect of comprehenders’ attitudes and emotional sensitivity to the topic of environmental issues is less evident for the rating of frequency because this self-report measure obscures the content of measurement (i.e., pro-environmental awareness) to a much greater extent than the more explicit self-report measure of seriousness.

## Experiment 2

The goal of Experiment 2 was to determine whether a moderating effect of environmental attitude found in Experiment 1 will stand up to empirical scrutiny in an experimental task that taps into automatic (rather than controlled) processes. To this end, we used a sentence-picture verification task in which participants read the same sentences as in Experiment 1, but this time their task was to decide whether the object shown in the subsequently presented picture was mentioned in the sentence. If automatic components of attitudes also affect language processing, then, similar to Experiment 1, the comprehender’s environmental awareness should moderate comprehension. Consequently, we should observe a significant Sentence Condition × Environmental Awareness interaction and/or a significant effect of Environmental Awareness.

Nonetheless, as discussed before, research on attitude formation suggests that measures of “explicit” and “implicit” attitudes often diverge in their results. Some studies found effects only on explicit measures (Gregg et al., 2006) while other studies found effects only on implicit measures (Olson and Fazio, 2006). Still others found effects on both explicit and implicit measures (Whitfield and Jordan, 2009). Such divergence between implicit and explicit tests in attitude formation research hints at the possibility that the results from a more “implicit” sentence-picture verification paradigm may also diverge from the results of a more “explicit” self-report questionnaire in the context of language comprehension research. One of the goals of Experiment 2 was thus to address this possibility.

### Method

#### Participants

One hundred and thirty-five native Portuguese-speaking university students took part in Experiment 2 in exchange for course credit. Because of the coronavirus disease 2019 (COVID-19), students signed up for a study online through the cloud-based Participant Management Software SONA. Stimulus presentation was controlled by a web-based service PsyToolkit, which was designed for setting up, running, and analyzing online reaction-time (RT) experiments (Stoet, 2010, 2017). The responses of seven participants were discarded for having accuracy <80% on the main task. Overall, the results of Experiment 2 are based on data from 128 participants (*M*_age_ = 24.47, *SD*_age_ = 7.04), of whom 93 were females.

#### Materials and Design

**FIGURE 2:**
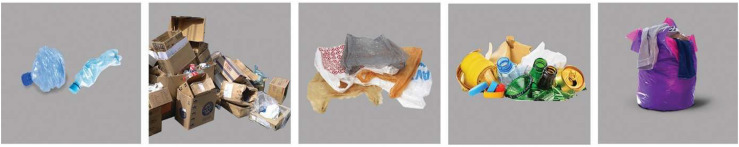
Samples of critical pictures used in Experiment 2.

The sentences and Environmental Attitudes Inventory were the same as in Experiment 1. Thirty-six colorful pictures were created to accompany the sentences. In order to ensure that the pictures depicted the environmental problems we intended them to, prior to experiment three independent raters determined whether the shown pictures matched the environmental problems described in the sentences. All raters stated that our pictures matched the sentences, thus ensuring that the pictured stimuli we used actually depicted the environmental problems/environmentally related objects we wanted them to depict. All pictures were of the same size (385 × 385 pixels) and depicted the environmental problem described in the preceding sentence on a gray background (see Figure 2)^2^.

#### Design and Procedure

The design was similar to Experiment 1, except that 36 pictures were added. Each participant saw 18 experimental sentence-picture pairs requiring “yes” responses and 18 filler sentence-picture pairs requiring “no” responses. Both experimental and filler sentences were identical in their format, thus making the potential strategy of selectively paying attention only to certain sentences suboptimal at best.

The procedure was similar to Experiment 1, except for the following important differences. First, the experimental flow was programmed in PsyToolkit web-based software (Stoet, 2010) that “forced” full screen mode on participants’ computers. Participants could not do the task using any kind of keyboardless device (e.g., a smartphone, a tablet, etc.) and a Safari web browser. Kim et al. (2019) experimentally tested the reliability of this online service in comparison to a lab-based service E-Prime 3.0 in a complex psycholinguistic task and found that the results from both software programs were similar. Second, the experiment began with six practice trials to ensure that participants understood the instructions of the sentence-picture verification paradigm. Each trial started with a fixation cross in the middle of a screen for 1000 ms. Then a sentence appeared at the center of the screen until participants pressed the Spacebar, thus indicating that they finished reading the sentence. After a spacebar press, the sentence was replaced by a fixation cross for 500 ms, immediately followed by a picture of an object that was either mentioned or not in the preceding sentence. Participants indicated their decision by pressing an “S” button for a “yes” response and an “N” button for a “no” response. Third, in the final part of the experiment participants indicated their level of agreement with 30 statements from the Portuguese version of the Environmental Attitudes Inventory on a 7-point scale from 1 (*Completely disagree*) to 7 (*Absolutely agree*).

#### Data Treatment

Prior to analysis, incorrect responses, filler items, and the data of participants with an overall accuracy <80% on the main task were excluded. Second, response times (RTs) were checked for normality using histograms with normal curve and Q-Q plots. RTs were positively skewed, and thus log10 transformation was applied to get normal distributions (Baayen, 2008). Finally, responses exceeding ±3 median absolute deviations (MAD) from the condition’s median were removed. ±3 MAD is a robust method of outlier treatment that is not affected by extremely high or extremely low values, and hence eliminates the need to define “arbitrary” lower and upper cutoff points (see Leys et al., 2013, for more information).

#### Statistical Analysis

All statistical analyses were performed in R by using the lme4 package (Bates et al., 2015) and lmerTest package (Kuznetsova et al., 2017) to obtain *p*-values. Mixed-effects logistic regression and linear mixed-effects models with random effects of participants and items were run on accuracy scores and RT data, respectively. For both accuracy and response times analyses, we first always fitted the full variance-covariance structure of random effects (the so-called “maximal” model; Barr et al., 2013). However, if the random effects structure was not supported by the data, we removed the most complex part of the random effects structure (see Matuschek et al., 2017, for a discussion of “model selection criterion”). The “maximal” model (Barr et al., 2013) for the present research contained sentence condition, environmental awareness score, as well as the interaction between the two as fixed effects; a by-participants random slope for sentence condition; and an intercept for participants and items. For the same reasons as in Experiment 1, no random slopes were specified for items and environmental awareness scores. If the “maximal” model failed to converge or produced a warning (e.g., a singular fit warning, which suggests that the model is overfitted) regarding the random effects structure, we first checked whether the model converges with a random effects structure for which no slope-intercept correlation term is specified. If this did not help, then we dropped a random slope in order not to (mistakenly) attribute all variability to the slope per participant instead of to the intercept (Brysbaert and Stevens, 2018). Finally, if one of the random intercepts was still found to be a redundant factor, then it was removed. With these considerations for random effects in mind, in the present research the best converging model for accuracy contained a random intercept for items and the best converging model for response times contained a random intercept for participants and items.

### Results and Discussion

#### Accuracy

**FIGURE 3:**
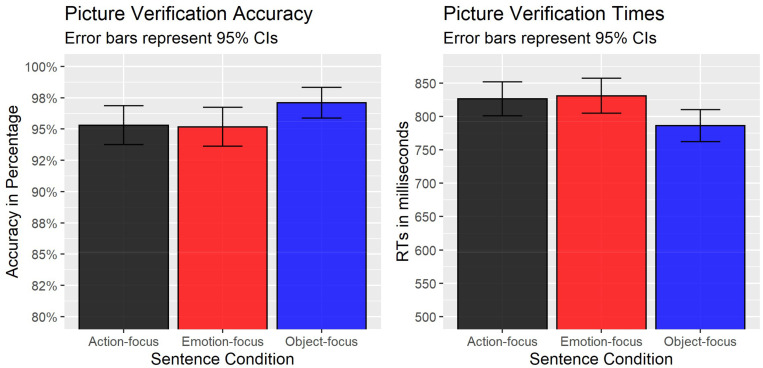
Mean accuracy and raw response times in Experiment 2.

Participants’ mean accuracy was high (95.9%). A likelihood ratio test of the best converging model (without warnings) with fixed effects of sentence condition, environmental concern, as well as the interaction between the two against the model with fixed effects of sentence condition and environmental concern showed no significant difference between the models [χ^2^(2) = 0.39, *p* = 0.821], thus suggesting that there was no evidence for the interaction between sentence condition and environmental concern. Therefore, the “simplified” model without an interaction term was used in the reporting of results. The results of the “simplified” model (Figure 3, left graph) showed a significant effect of “emotion-focus” (estimate = −0.58, *SE* = 0.28, *z* = −2.04, *p* = 0.042, odds ratio = 0.56) condition and a trending effect of “action-focus” (estimate = −0.52, *SE* = 0.29, *z* = −1.81, *p* = 0.070, odds ratio = 0.60) condition, reflecting the fact that accuracy in the “action-focus” (95%) and “emotion-focus” (95%) conditions was lower than in the referent “object-focus” (97%) condition. Finally, there was no effect of participants’ environmental concern (estimate = 0.11, *z* = 1.19, *p* = 0.234, odds ratio = 1.14), which suggests that more environmentally concerned participants were not significantly more accurate in their responses than less environmentally concerned participants.

#### Response Times (RTs)

Similar to accuracy scores, a likelihood ratio test showed no significant difference between the models with the interaction term and without the interaction term [χ^2^(2) = 2.39, *p* = 0.303], thus suggesting that the interaction between sentence condition and environmental concern was not significant. Thus, the “simplified” model without the interaction term was used in the reporting of results. The analyses demonstrated that participants’ response times were positively correlated with corresponding effects in the accuracy scores, thus precluding speed-accuracy tradeoffs. More specifically, as shown in Figure 3 (right graph), there was a significant effect of “emotion-focus” (estimate = 0.02, *SE* = 0.01, *t* = 2.81, *p* = 0.005) and “action-focus” (estimate = 0.02, *SE* = 0.01, *t* = 2.80, *p* = 0.005) conditions, reflecting the fact that RTs in the “action-focus” and “emotion-focus” conditions were slower than in the referent “object-focus” condition (see Figure 3). Finally, similar to accuracy analysis, there was no effect of participants’ environmental concern (estimate = 0.01, *SE* = 0.01, *t* = 0.84, *p* = 0.404) on RTs.

Thus, this pattern of results accords with the possibility outlined earlier: environmental problems are kept highly accessible in the “object-focus” condition (similar to results from Experiment 1), but comprehenders’ implicit (as compared to explicit) attitudes seem to have no significant effect on situation model construction and language comprehension processes.

## General Discussion

The present research was motivated by three goals. The first goal was to examine whether focusing readers’ attention differently on the protagonist dimension would affect the accessibility of environmental problems described in the sentence. The second goal was to begin documenting the contributions of such unique comprehender characteristics as pro-environmental attitudes to interpretation of linguistic input. The third goal was to explore in what task situations comprehenders’ pro-environmental awareness may modulate language comprehension processes. To this end, we asked participants to read sentences about environmental issues that focused on different mental and physical attributes (i.e., emotional state, action state, etc.) of the same entities (protagonists and objects) and then self-report their own thoughts, feelings, and behaviors on the topic of environment by responding to the items from the Environmental Attitudes Inventory. Importantly, we manipulated the task requirements across two experiments by administering a self-report task (Experiment 1), which required the participants to rate the seriousness and the frequency of the problem mentioned in a sentence; and administering a sentence-picture verification paradigm (Experiment 2), which required the participants to merely indicate if the object depicted in the picture (related to a certain environmental problem) was mentioned in the preceding sentence. The results of these experiments suggest that the focus of a sentence on the environmental problem (i.e., “object-focus” condition) rather than the protagonist’s state (i.e., “emotion-focus” and “action-focus” conditions) enhances accessibility of the information related to environmental issues (e.g., plastic garbage); that the comprehender’s level of environmental awareness influences one’s attention during language processing; and that comprehender characteristics significantly modulate comprehension processes only when the measure taps into conscious representations.

The findings reported in this article add to the empirical evidence that comprehenders track various dimensions of situation models during language comprehension (e.g., Gernsbacher et al., 1992; Zwaan et al., 1995a,b; de Vega, 1996; Rinck and Bower, 2000; Rinck et al., 2003; Therriault et al., 2006; Kang et al., 2019); and that the comprehenders’ situational models capture information about a character’s emotional and actions states (e.g., de Vega, 1996; Borghi, 2004; Horchak et al., 2016; Horchak and Garrido, 2020a). Whereas most previous studies focused their efforts on understanding how multiple dimensions of the situation model (e.g., protagonists, intentionality, causation, etc.) are constructed and updated during language processing (Magliano and Schleich, 2000; Rich and Taylor, 2000; Rapp et al., 2001; Rinck and Weber, 2003), the present research assessed the extent to which focusing participants’ attention on the entity’s physical and mental attributes (i.e., a protagonist dimension) influences situation model construction. Our results show that when a sentence focused readers’ attention on protagonists’ emotional and action states (compared to when the attention was on the target environmental problem), then participants’ ratings were lower (Experiment 1) and response times to the picture probes (Experiment 2) were longer. These additive effects on ratings and response times are exactly what one would expect to observe in support of the hypothesis that readers direct their focus of attention to those aspects of the event that they consider to be in the spotlight for the current situation model. Our explanation of these results assumes that participants were building situation models organized around (1) a target environmental problem in the “object-focus” condition, (2) a protagonist’s mental state in the “emotion-focus” condition, and (3) a protagonist’s physical state in the “action-focus” condition. The experiments required from participants to either evaluate the seriousness and the frequency of environmental problem or verify if the environmentally related object (e.g., plastic bottle) was mentioned in the sentence, and therefore the greater was the number of “irrelevant” facts associated with a given problem, the more difficult it was for participants to retrieve the relevant fact. Thus, comprehenders “follow” not only the major character and objects mentioned in the sentence, but also their mental and physical attributes. The accessibility of the objects mentioned in the critical sentence “fades” as the focus moves on to other aspects of the described event.

The present research represents a significant departure from traditional research on situation model construction as it also assessed the relevance of such comprehender characteristics as pro-environmental attitudes for sentence processing. Although some accounts of conceptual processing and language comprehension addressed how visual and action contexts affect language processing mechanisms (Zwaan, 2004; Knoeferle and Crocker, 2006; Altmann and Kamide, 2007; Altmann and Ekves, 2019), they have not tended to focus on how social evaluation may guide comprehension processes. Our findings fill this gap by integrating the insights from social psychological research on automatic and controlled components of attitudes with cognitive psychology research on situation model construction. More specifically, we asked in what task situations comprehenders’ environmental awareness may mediate attentional focus during situation model construction. Our data suggest a more complex relation between language processing mechanisms and the characteristics of the comprehender than one could have predicted with confidence. Interestingly, the influence of comprehender characteristics on language processing seems to depend more on whether the measure aims to capture automatic or controlled components of attitudes than the measure itself. Such a conclusion follows from the results showing no moderating effect of comprehenders’ pro-environmental attitudes on the ratings of frequency (“explicit” task in Experiment 1) and response times to picture probes (“implicit” task in Experiment 2). That is, while ratings of frequency and response times to picture probes are radically different types of tests, what both have in common is that they attempt to measure pro-environmental attitudes in a more automatic fashion, thus considerably reducing participants’ ability to control their responses in line with social desirability. Indeed, participants’ pro-environmental attitudes only moderated the more explicit rating of seriousness (“explicit” task in Experiment 1): the information about the environmental problem was equally accessible in all sentence conditions for participants with low environmental awareness, but not for participants with high environmental awareness. These findings thus support a conclusion that the influence of comprehenders’ pro-environmental attitudes on language processing depends on whether automatic or controlled factors affect social evaluation and not the directness or indirectness of the test itself (see Ranganath et al., 2008, for a related discussion).

Given the pattern of the results observed, the obvious question is why automatic components of attitudes did not exhibit moderating effects on language comprehension processes in our research. This question is of great empirical and theoretical importance as most psycholinguistic tasks do not require introspection (i.e., the examination of one’s own conscious thoughts and feelings) for the assessment of psychological attributes. Indeed, at this point it is difficult to say with any precision in what situations implicit attitudes moderate comprehension processes, but what seems to have occurred in the present research is that participants’ unconscious reaction toward environmental issues was lagging behind their conscious desire for environmental improvement, which is consistent with a model of dual attitudes (Wilson et al., 2000). The central idea underlying this model is that previously formed representations cannot be easily erased from memory when people learn new facts about something (e.g., environment is a really big problem) and then integrate them with old inconsistent information (e.g., environment is as important as many other problems) that reflects what they previously believed in. As nicely put by Gregg et al. (2006), highly stable, old representations may be compared to a credit card debt and excess calories that are difficult to cast aside. When people are faced with a certain stimulus, their conscious interpretation of it is supplemented by an automatic reaction. However, once the attitude toward a stimulus is formed through multiple direct experiences, attempts to subsequently override this attitude with a newly formed one will be successful to the extent to which the recently acquired knowledge is learned.

The present research has at least two limitations. The first limitation is that we assessed only those attitudes that are related to environmental awareness. It is thus unclear if the results would change if we considered attitudes that are more strongly related to socially sensitive topics (e.g., racial discrimination). For example, a meta-analysis of Greenwald et al. (2009) revealed that there is a considerable body of research showing an impact of old and recent experiences on both explicit and implicit measures, especially with regards to domains of stereotyping and prejudice. Furthermore, the effects of such characteristics of the comprehenders as age, education level, and knowledge of foreign languages, were successfully detected in language comprehension tasks using more implicit measurement methods, such as eye tracking (Huettig et al., 2011; Mishra et al., 2012; Carminati and Knoeferle, 2013, 2016; Ito et al., 2018). The second limitation is that the paradigms used in the present research do not provide a strong test between a propositional network view (e.g., Bower and Rinck, 2001) and a situation model view (e.g., Radvansky et al., 1998) that may explain the nature of the mental representation used to perform the tasks. For example, it was demonstrated that a sentence-picture verification task reveals the contents of mental representations that are activated as comprehenders read sentences, but does not provide a strong test for the claim that sensorimotor processes contribute functionally to language comprehension (e.g., Ostarek et al., 2019; Horchak and Garrido, 2020b). Therefore, it is not clear whether symbolic representations somehow interacted with sensorimotor (embodied) representations when participants were processing the test sentences in the current research (see Horchak et al., 2014, for a review of literature on contribution of symbolic and embodied representations to language comprehension). Although the present studies were not designed to address these issues, we believe that there are good reasons to believe that the propositional information was, at the very least, complemented with situational information beyond that provided by the sentence. Why would the focus on protagonists’ mental attributes, for instance, make a difference relative to the focus on the target object in a sentence? Recall that emotion-focused sentences received similar ratings of seriousness when compared to the object-focused sentences. Perhaps it is because participants processed not only an explicitly mentioned emotional state of the protagonist (e.g., “John *got upset* with the garbage on the beach”), but also a few other automatic inferences, such as “Environment is important for John,” “John must be sad right now,” “John must not like people polluting the beach,” etc. These kinds of inferences likely caused mental attributes of the protagonist (on the one hand) and the environmentally related target object (on the other hand) compete for status as the concept by which the situation model is organized.

An important qualification of the present research is that it does not constitute direct evidence for the claim that mental representations of comprehenders were always organized in terms of real-world situations while they were reading the test sentences. It could be argued, for example, that a target word’s meaning was simply represented in more detail when it was focused in a sentence’s information structure (see Birch and Rayner, 1997; Sturt et al., 2004; Spalek et al., 2014; Gotzner et al., 2016; and Yang et al., 2019, for empirical evidence on linguistic focus hypothesis). Indeed, all our sentences were of the structure “Subject–Verb–Object” and participants’ assessments of an environment issue (e.g., plastic rubbish, raised water level, mud from rains) could merely depend on whether the subject (i.e., a protagonist that experienced a particular action or emotion) or the object (i.e., an environmental issue) was in the focus of the sentence as defined by verb information. That is, focused information could have a privileged memory representation as a result of the governance of linguistic constructions. Although this scenario is consistent with the results of Experiment 2, our data from Experiment 1 have also shown that participants did not consider this as the only way to organize the information. If linguistic factor was the only one to guide attention to different aspects of the referential situation, then we should not have observed the following results: (1) participants with higher concern over environmental issues (from a test of attitudes) gave overall higher ratings; (2) object-focused sentences received similar ratings of seriousness when compared to the emotion-focused sentences; and (3) action-focused sentences received lower ratings of seriousness than object-focused sentences only with regards to the participants with higher concern over environmental issues. Given the above evidence, our findings constitute evidence that situation-based representations, at the very least, had to complement gist representations for adequate comprehension.

The remaining discussion will be focused on the following two aspects. First, we will discuss how the “Dynamic Text Comprehension (DTC)” framework of Rapp and van den Broek (2005) relates to the theoretical position advocated by this paper, namely that comprehenders’ attention to events can be driven by task instructions and comprehenders’ goals. Second, based on this discussion, we will provide a putative explanation of how comprehenders’ could organize their representations around situation principles while reading the test sentences used in the present research.

According to the DTH framework, comprehending a text is tantamount to the construction of a situation model, whereby readers are able to not just understand the exact content of the text, but also infer implicit information (i.e., that was not directly stated in the text). Importantly, however, DTH proposes that successful comprehension arises from the interactive contributions of three factors: a text (e.g., text difficulty, genre, etc.), a reader (e.g., prior knowledge, cognitive abilities, etc.), and a task (e.g., instructions, task difficulty, etc.). To demonstrate this, Rapp and Kendeou (2007) explored how particular methodologies used in “online” and “offline” comprehension tasks differentially modulate readers’ attention. More specifically, participants were asked to read short stories implying a character’s potential traits (e.g., “Albert’s room is messy” ▶ Albert is sloppy) that ended with (1) a *simple refutation* of that trait (e.g., “Albert cared about the condition of his room” ▶ Albert is organized); (2) a more *explanatory refutation* explaining why an inferred trait might be wrong (e.g., “Albert cared about the condition of his room, but had only moved in to the house yesterday” ▶ Albert is organized); or (3) further *support* for that trait (e.g., “Albert’s room is messy. He has some dirty laundry” ▶ Albert is sloppy). The researchers administered two types of tasks. In the “online” comprehension task participants read each sentence, one at a time, with reading times recorded for the outcome of the story (i.e., a part that either supported or refuted the initially described character trait). In the “offline” comprehension task participants were asked to explicitly indicate whether they agreed or disagreed with the outcome of the story. A key research question was whether comprehenders would update a character trait as a function of the information provided by the refutation. Rapp and Kendeou (2007) found that for *simple* refutations participants took longer to read trait-inconsistent than consistent outcomes, but for *explanatory* refutations participants took longer to read trait-consistent than inconsistent outcomes. They interpreted this finding as providing support for the claim that readers no longer expected characters to behave in trait-consistent ways when sufficient information (i.e., a condition of *explanatory* refutation) for that trait was provided. Perhaps even more interestingly, the results from “offline” task showed that the updating of trait information was observed for both types of refutations (i.e., participants always agreed that a previously encoded trait was incorrect), thus suggesting that “online” and “offline” tasks may encourage participants to focus on different aspects of a scene as a function of methodologies. In support of this is also other empirical evidence showing, for instance, that recall tasks encourage comprehenders to focus on the task at hand while ignoring prior knowledge, but judgments tasks encourage careful examination of the accuracy of information based on both what was read and what was previously experienced (e.g., Egidi and Gerrig, 2006).

The aforementioned theoretical and empirical evidence suggests that language comprehension arises not only from what a text contains, but also from task instructions and a comprehender’s interest in a topic (as defined by world knowledge or beliefs). If we accept that the interaction of these factors may indeed encourage different profiles of a comprehender’s attention, then it is reasonable to assume that the assumptions from DTH framework can also be extrapolated to the present research. More precisely, for the current experiments we may consider focus condition as a major sentence factor; the methodology as a major task factor; and a comprehender’s level of environmental concern as a major reader factor. On this account, differences in methodologies between a questionnaire and a sentence-picture verification paradigm, either explicitly or implicitly, could encourage readers to process information presented in a sentence differently. By using a self-report measure (a questionnaire) of seriousness, participants were explicitly asked to evaluate whether a sentence described a serious situation related to environment. It should come as no surprise then that the more the sentence aligned with participants’ beliefs, the more predisposed they were to consider the sentence information carefully. Indeed, comprehenders particularly sensitive to environmental issues could focus not only on what was within linguistic focus (e.g., environmental issues), but also on what was within the focus of their own feelings or beliefs, such as the feeling that they should be doing more to help the environment, thus lingering on content such as “cried about” or “hated.” Perhaps, then, it was precisely because of this why emotion-focused sentences received similar ratings of seriousness when compared to the object-focused sentences.

By using a self-report measure of frequency, in contrast, the true intent of a question (i.e., participants’ *real* level of environmental concern) was obfuscated. Yet participants had enough time to carefully evaluate their level of agreement or disagreement with the information in the sentence. Notably, a self-report measure of frequency was effective at discouraging responses in line with social desirability because participants’ evaluations of the environmental problems were now consistent with our predictions: ratings were lower when sentences focused on protagonist emotion or action, rather than the sentence object. But it is worth noting that the evidence of comprehenders’ concern over environmental issues (i.e., higher concern = higher ratings; lower concern = lower ratings) was clearly observed for both ratings of seriousness and frequency.

Finally, by using a sentence-picture verification paradigm, participants were almost entirely discouraged from consciously evaluating the information in any particular way (e.g., if it matches their beliefs), and hence could easily adopt a strategy to focus on the most important information in a sentence, precisely because it was more relevant for completing the task (see Rapp and Mensink, 2011, for further discussion). As such task did not foster careful evaluation, it remains possible that participants primarily relied on the governance of linguistic constructions, which maintained focus on either the protagonist or sentence object (i.e., environmental problem). Whenever linguistic focus was on the environmental problem in a sentence (i.e., sentence object), participants’ verification times of environment-related objects were faster.

Admittedly, our explanation of the observed pattern of results requires additional empirical support to further scrutinize how unique comprehenders’ characteristics and task requirements influence sentence processing. It is our hope that our research will contribute to an agenda of items that merit discussion and future investigation to help us further develop theoretical accounts that assess the role of speaker and comprehender characteristics for situated language comprehension (e.g., Social Coordinated Interplay Account; Münster and Knoeferle, 2018).

According to our present analysis, unique comprehenders’ characteristics such as attitudes help predict the variability of context effects during sentence processing. However, it is unlikely that such characteristics may affects all kinds of information. The present research supports this claim in light of the results showing that the action-focused sentences were not so heavily moderated by attitudes. At this point in time it is hard to say with any precision why this happened. Findings from the literature on the action-attitude gap in environmental psychology provide some clues in this regard. More specifically, they suggest that the lack of a significant effect may be explained by constraints of behavioral control (Koger and Winter, 2010). According to this framework, while people report having sustainable behaviors toward the environment, their behavioral intention to actually act “ecologically” may lag behind the belief that this would only have a minimal impact on the environment. Thus, whereas it may be intuitive that the seriousness of the described environmental problem should be accessible in all sentence conditions (albeit to varying degrees), the focus of a sentence on the action might lead to questioning the validity of the facts (e.g., cleaning garbage on the beach is a waste of time as environmentally irresponsible behaviors outweigh sustainable behaviors). It remains to be seen, however, whether these predictions hold true in the task used to study language comprehension processes.

To conclude, the present research suggests that comprehenders’ attitudes may alter how they focus on the major character and track his/her physical and mental attributes during sentence comprehension. Comprehenders’ implicit attitudes (as compared to explicit ones) may create a stumbling block for investigating the role of comprehender characteristics during language comprehension, and hence the use of varied measurement procedures is warranted. Future research can explore to a much greater extent how attitudes related to more sensitive topics (e.g., prejudice and discrimination) affect language comprehension processes over the course of processing the sentence (e.g., eye tracking or word-by-word sentence design) to be able to better understand the functional mechanisms behind the obtained results.

## Data Availability Statement

The datasets presented in this study can be found in online repositories. The names of the repository/repositories and accession number(s) can be found below: https://osf.io/qbgsd/?view_only=d6cd8cc712aa4c69bcf3ea6ecb06128f.

## Ethics Statement

Ethical review and approval was not required for the study on human participants in accordance with the local legislation and institutional requirements. The patients/participants provided their written informed consent to participate in this study.

## Author Contributions

OH and MG idealized the study. OH and MG designed the sentence stimuli. OH designed the picture stimuli and drafted the manuscript. OH and MG involved in acquisition of data, analysis and interpretation of data, critical revision of the manuscript, and approval of the submitted version for publication. Both authors contributed to the article and approved the submitted version.

## Conflict of Interest

The authors declare that the research was conducted in the absence of any commercial or financial relationships that could be construed as a potential conflict of interest.

## Footnotes

1 Ratings for one item (about organic farming) were removed from the analysis as the average item rating was extremely low (2 out of 10). This indicates that participants did not believe that the sentence described a serious environmental problem.

2 To allow for accurate comparison of results between Experiments 1 and 2, we removed the same item (i.e., about organic farming) as in Experiment 1 from the analysis.
