# Supplementary material for: Explicit (Not Implicit) Attitudes Mediate the Focus of Attention During Sentence Processing
Source: Front Psychol. 2020 Dec 23;11:583814. doi: 10.3389/fpsyg.2020.583814 (PMC7786004; doi:10.3389/fpsyg.2020.583814)
Supplement: Supplementary file 1 [file Data_Sheet_1.docx]

**APPENDIX A**

Critical sentences used in Experiments 1 and 2 in three different conditions

(an approximate translation from Portuguese into English*)

1. John *observed / stepped on / got irritated* *with* the plastic bottles on the floor.
2. Mary *noticed / picked up / got irritated with* the plastic bags in the sea.
3. Tania *pondered about / picked up / got angry with* the quantity of new clothes left in the garbage can.
4. Mary *noted / jumped to protect herself from / got scared of* the increase in the water level in a dam.
5. Mary *saw / took / hated* the cream tested on animals.
6. Tania *reflected on / folded / cried about* the petition about extinction of pandas.
7. John *got irritated* *with* */ observed / stepped* on the hose wasting water.
8. Mary *got irritated with / noticed / picked up* the garbage on the beach.
9. Tania *got angry with / pondered about / folded* the carton boxes piled up on the street.
10. Mary *got scared of / noticed / jumped close to* the abandoned dog.
11. Pedro *hated / saw / ran on* the mud caused by rains.
12. Tania *cried about / reflected about / climbed the tree in protest* *over* cutting of trees.
13. John *stepped on / got irritated with / observed* the bottle of herbicide.
14. Pedro *picked up / hated / noticed* the tossed take out plastic containers.
15. Mary *jumped over the fence to protect / got scared of / saw* an animal in danger of extinction.
16. Tania *took / cried of seeing / reflected on* the caged dogs.
17. John *ran away from / got irritated with / observed* the bull fighting.

*Note:* Note that the original sentences were in Portuguese (European), and hence the approximate English translation may not always sound natural due to language differences.

**APPENDIX B**

Items taken from the original version (Milfont & Duckitt, 2010) of the Environmental Attitudes Inventory (and validated for the Portuguese sample by Domingues & Gonçaçves, 2018) used to assess participants’ level of environmental awareness.

1. I really like going on trips into the countryside, for example to forests or ﬁelds.
2. Being out in nature is a great stress reducer for me.
3. I enjoy spending time in natural settings just for the sake of being out in nature.
4. Industry should be required to use recycled materials even when this costs more than making the same products from new raw materials.
5. People in developed societies are going to have to adopt a more conserving life-style in the future.
6. I don’t think people in developed societies are going to have to adopt a more conserving life-style in the future.
7. I would NOT get involved in an environmentalist organization.
8. Environmental protection costs a lot of money. I am prepared to help out in a fund-raising effort.
9. I would like to support an environmental organization.
10. The worst thing about the loss of the rain forest is that it will restrict the development of new medicines.
11. One of the most important reasons to keep lakes and rivers clean is so that people have a place to enjoy water sports.
12. We need to keep rivers and lakes clean in order to protect the environment, and not as places for people to enjoy water sports.
13. If things continue on their present course, we will soon experience a major ecological catastrophe.
14. Humans are severely abusing the environment.
15. I do not believe that the environment has been severely abused by humans.
16. I’d prefer a garden that is wild and natural to a well-groomed and ordered one.
17. I’d much prefer a garden that is well groomed and ordered to a wild and natural one.
18. Grass and weeds growing between pavement stones really looks untidy.
19. I could not be bothered to save water or other natural resources.
20. In my daily life I try to ﬁnd ways to conserve water or power.
21. Whenever possible, I try to save natural resources.
22. Humans were meant to rule over the rest of nature.
23. Human beings were created or evolved to dominate the rest of nature.
24. I do not believe humans were created or evolved to dominate the rest of nature.
25. Humans do NOT have the right to damage the environment just to get greater economic growth.
26. We should no longer use nature as a resource for economic purposes.
27. The question of the environment is secondary to economic growth.
28. I do not believe protecting the environment is an important issue.
29. I do not believe nature is valuable for its own sake.
30. I don’t get upset at the idea of forests being cleared for agriculture.

**APPENDIX C**

The results of the “final” Cumulative Link Mixed Model for ratings of seriousness and frequency

| Fixed Effects (Seriousness) | | | | | | | |
| --- | --- | --- | --- | --- | --- | --- | --- |
|  | Beta | SE | Odds Ratios | | 95% CI for Odds Ratios | z | p |
| Emotion-focused condition | -0.14 | 0.12 | 0.87 | | 0.69 – 1.10 | -1.17 | .244 |
| Action-focused condition | -0.79 | 0.11 | 0.45 | | 0.36 – 0.56 | -7.00 | <.001 |
| Environmental Concern | 0.52 | 0.15 | 1.68 | | 1.26 – 2.23 | 3.56 | <.001 |
| Emotion ∗ Environmental Concern | 0.09 | 0.12 | 1.09 | | 0.86 – 1.37 | 0.72 | .470 |
| Action ∗ Environmental Concern | -0.24 | 0.11 | 0.79 | | 0.63 – 0.98 | -2.13 | .033 |
| Random Effects (Seriousness) | | | | | | | |
|  | Variance | S.D. | | | | Correlation | |
| Participants (Intercept) | 1.69 | 1.30 | | | |  | |
| Items (Intercept) | 0.32 | 0.57 | | | |  | |
| Emotion-focused condition (slope) | 0.32 | 0.57 | | | | 0.22 | |
| Action-focused condition (slope) | 0.16 | 0.40 | | | | -0.63 | |
| Model fit (Seriousness) | | | | | | | |
| R^2^ | Marginal |  | | Conditional R^2^ | | | |
|  | 0.06 |  | | 0.42 | | | |
| Key: Cumulative Link Mixed Model fitted with the Laplace approximation  R Code*: Seriousness ~ Sentence Condition + Environmental Concern + Sentence Condition : Environmental Concern + (1 + Sentence Condition \| participants) + (1 \| items) | | | | | | | |
| Fixed Effects (Frequency) | | | | | | | |
|  | Beta | SE | Odds Ratios | | 95% CI for Odds Ratios | z | p |
| Emotion-focused condition | -0.23 | 0.10 | 0.80 | | 0.65 – 0.97 | -2.24 | .025 |
| Action-focused condition | -1.17 | 0.11 | 0.31 | | 0.25 – 0.39 | -10.39 | <.001 |
| Environmental Concern | 0.27 | 0.12 | 1.31 | | 1.04 – 1.66 | 2.33 | .020 |
| Random Effects (Frequency) | | | | | | | |
|  | Variance | S.D. | | | | Correlation | |
| Participants (Intercept) | 1.31 | 1.15 | | | |  | |
| Items (Intercept) | 0.54 | 0.74 | | | |  | |
| Emotion-focused condition (slope) | 0.03 | 0.18 | | | | 0.74 | |
| Action-focused condition (slope) | 0.19 | 0.44 | | | | -0.25 | |
| Model fit (Frequency) | | | | | | | |
| R^2^ | Marginal |  | | Conditional R^2^ | | | |
|  | 0.06 |  | | 0.41 | | | |
| Key: Cumulative Link Mixed Model fitted with the Laplace approximation  R Code*: Frequency ~ Sentence Condition + Environmental Concern + (1 + Sentence Condition \| participants) + (1 \| items) | | | | | | | |

*Note*. For convenience of statistical analyses, variable names were shortened in the data file.

**APPENDIX D**

The results of the “final” Generalized Linear Mixed Model and Linear mixed model for Accuracy and RTs, respectively

| Fixed Effects (Accuracy) | | | | | | | |
| --- | --- | --- | --- | --- | --- | --- | --- |
|  | Beta | SE | Odds Ratios | | 95% CI for Odds Ratios | z | p |
| Intercept | 4.16 | 0.39 | 64.31 | | 30.05 – 137.6 | 10.73 | <.001 |
| Emotion-focused condition | -0.58 | 0.28 | 0.56 | | 0.32 – 0.98 | -2.04 | .041 |
| Action-focused condition | -0.52 | 0.29 | 0.60 | | 0.34 – 1.04 | -1.81 | .070 |
| Environmental Concern | 0.13 | 0.11 | 1.14 | | 0.92 – 1.41 | 1.19 | .234 |
| Random Effects (Accuracy) | | | | | | | |
|  | Variance | S.D. | | | | Correlation | |
| Items (Intercept) | 1.38 | 1.17 | | | |  | |
| Model fit (Accuracy) | | | | | | | |
| R^2^ | Marginal |  | | Conditional R^2^ | | | |
|  | 0.02 |  | | 0.31 | | | |
| Key: Generalized linear mixed model fit by maximum likelihood (Laplace Approximation)  R Code*: Accuracy ~ Sentence Condition + Environmental Concern + (1 \| items) | | | | | | | |
|  | | | | | | | |
| Fixed Effects (RTs) | | | | | | | |
|  | Beta | SE | 95% CI | | | t | p |
| Intercept | 2.87 | 0.02 | 2.84 – 2.91 | | | 161.58 | <.001 |
| Emotion-focused condition | 0.02 | 0.01 | 0.01 – 0.03 | | | 2.81 | .005 |
| Action-focused condition | 0.02 | 0.01 | 0.01 – 0.03 | | | 2.80 | .005 |
| Environmental Concern | 0.01 | 0.01 | -0.01 – 0.03 | | | 0.84 | .404 |
| Random Effects (RTs) | | | | | | | |
|  | Variance | S.D. | | | | Correlation | |
| Participants (Intercept) | 0.01 | 0.11 | | | |  | |
| Items (Intercept) | 0.00 | 0.06 | | | |  | |
| Model fit (RTs) | | | | | | | |
| R^2^ | Marginal |  | | Conditional R^2^ | | | |
|  | 0.01 |  | | 0.53 | | | |
| Key: Linear mixed model fit by REML.T-tests use Satterthwaite's method  R Code*: RT ~ Sentence Condition + Environmental Concern + (1 \| participants) + (1 \| items) | | | | | | | |

*Note*. For convenience of statistical analyses, variable names were shortened in the data file.
